# Multiple Fenestrated Dural Venous Sinuses: A Case Report

**DOI:** 10.7759/cureus.70766

**Published:** 2024-10-03

**Authors:** Keaton N Ott, Rarinthorn Samrid, Yoko Tabira, Kazzara T Raeburn, Kathleen C Bubb, Aaron S Dumont, Joe Iwanaga, R. Shane Tubbs

**Affiliations:** 1 School of Medicine, Tulane University, New Orleans, USA; 2 Department of Anatomy, Khon Kaen University, Khon Kaen, THA; 3 Department of Anatomy, Kurume University School of Medicine, Kurume, JPN; 4 Anatomical Sciences, St. George's University, St. George's, GRD; 5 Anatomical Sciences, Weil-Cornell Medicine, New York, USA; 6 Department of Neurosurgery, Tulane Center for Clinical Neurosciences, Tulane University School of Medicine, New Orleans, USA; 7 Neurosurgery and Ochsner Neuroscience Institute, Ochsner Health, New Orleans, USA

**Keywords:** anatomy, cranial, imaging, skull, variations, veins

## Abstract

Variations of the intradural venous sinuses are common. However, to the best of our knowledge, multiple fenestrated sinuses in the same specimen have not previously been reported. Herein, we report the cadaveric findings of fenestrations found in the left and right transverse and superior sagittal sinuses (SSS). The right transverse sinus was more superiorly located compared to its left counterpart. No occipital sinus was identified, and the straight sinus drained near the proximal left transverse sinus. The right transverse sinus was dominant and slightly larger in diameter throughout its course. Variation in the dural venous system is essentially a rule rather than an exception; however, configurations of these structures can be used as anatomical landmarks, making knowledge of potential anatomical variations of the dural venous sinuses critical for precluding complications during endovascular or neurosurgical procedures. The embryological development of these sinuses is also discussed.

## Introduction

The dural venous sinuses are valveless venous channels that serve as the final collecting system of blood from the brain and extracranial venous system before draining into the internal jugular veins. Fed by protruding arachnoid granulations, these channels are the primary sites of cerebrospinal fluid absorption [1]. They include the superior and inferior sagittal, straight, two transverse, two sigmoid, occipital, two cavernous, two intercavernous, superior and inferior petrosal, two sphenoparietal, basilar, and marginal sinuses [1].

Embryological development of the dural venous sinuses suggests how variations of these structures could arise [2]. The first true drainage channels of the head, the primary head veins, receive many tributaries, each aggregating into three vascular plexuses: anterior, middle, and posterior [2]. The dural venous sinuses are formed from the vascular mesh of these plexuses. For example, the superior and inferior sagittal sinuses arise from merging tributaries of the anterior and middle dural plexuses. These merging tributaries create a series of lakelets forming the sagittal plexus. This midline plexiform channel dips between the cerebral hemispheres, giving rise to the superior and inferior sagittal sinuses [2,3]. This mesh-like development of the superior sagittal sinus (SSS) suggests how malformations could develop and why a plexiform arrangement of the sinus can persist into adulthood [3].

As the stalk of the original anterior dural plexus disappears and the anterior and middle dural plexuses are further integrated, a new venous channel develops in the fetal brain. This channel, which also receives a connection from the posterior dural plexus and thus drains all three plexuses, is the primitive transverse sinus [2,4]. As the growing cerebral hemispheres and cerebellum form and compress the tentorium cerebelli, the transverse sinus lying within, once vertically aligned with the developing internal jugular vein, elongates anteroposteriorly [4]. During the 17th to 18th fetal weeks, the transverse sinus balloons from its lateral border medially toward the primitive torcular Herophili [5]. It enlarges until a relatively even caliber is attained at six to seven fetal months [5].

The dural venous system is inherently asymmetrical. For example, in most adults, the superior sagittal sinus drains mainly into the right transverse sinus, and the straight sinus drains mostly into the left one [1,6]. Owing to their embryological development from a plexiform venous network, the dural venous sinuses and their connecting veins vary considerably among individuals [1]. Numerous studies have investigated the anatomical variations/malformations of the dural venous sinuses [7,8].

This paper discusses an unusual case of multiple dural sinus fenestrations.

## Case presentation

**Figure 1:**
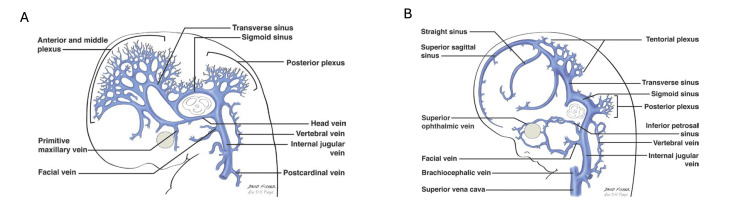
Embryological development of the dural venous sinuses. (A) Approximately day 48, and (B) approximately 9th week. Note the progression of the anterior, middle, and posterior venous plexuses from a multiply fenestrated venous network to a less network-like configuration between the left and right images (Courtesy Professor R. Shane Tubbs).

**Figure 2:**
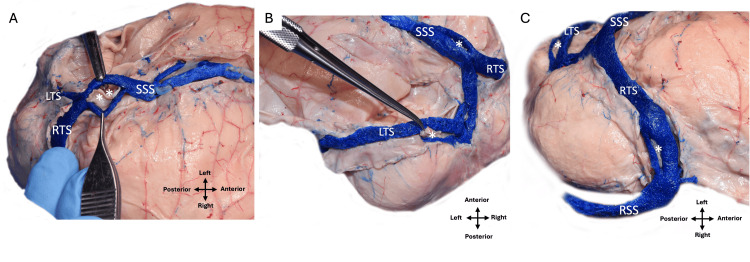
Cadaveric dissection of the case described herein. (A) Superior view noting the fenestrated superior sagittal sinus (SSS) (**) and left (LTS) and right (RTS) transverse sinuses. (B) Left lateral view noting the fenestrated left (LTS) (*). Also note the RTS and fenestrated (*) SSS. (C) Right lateral view noting a fenestrated (*) RTS. Note the fenestrated (*) LTS. Observe the SSS and right sigmoid (RSS) sinuses for reference.

**Figure 3:**
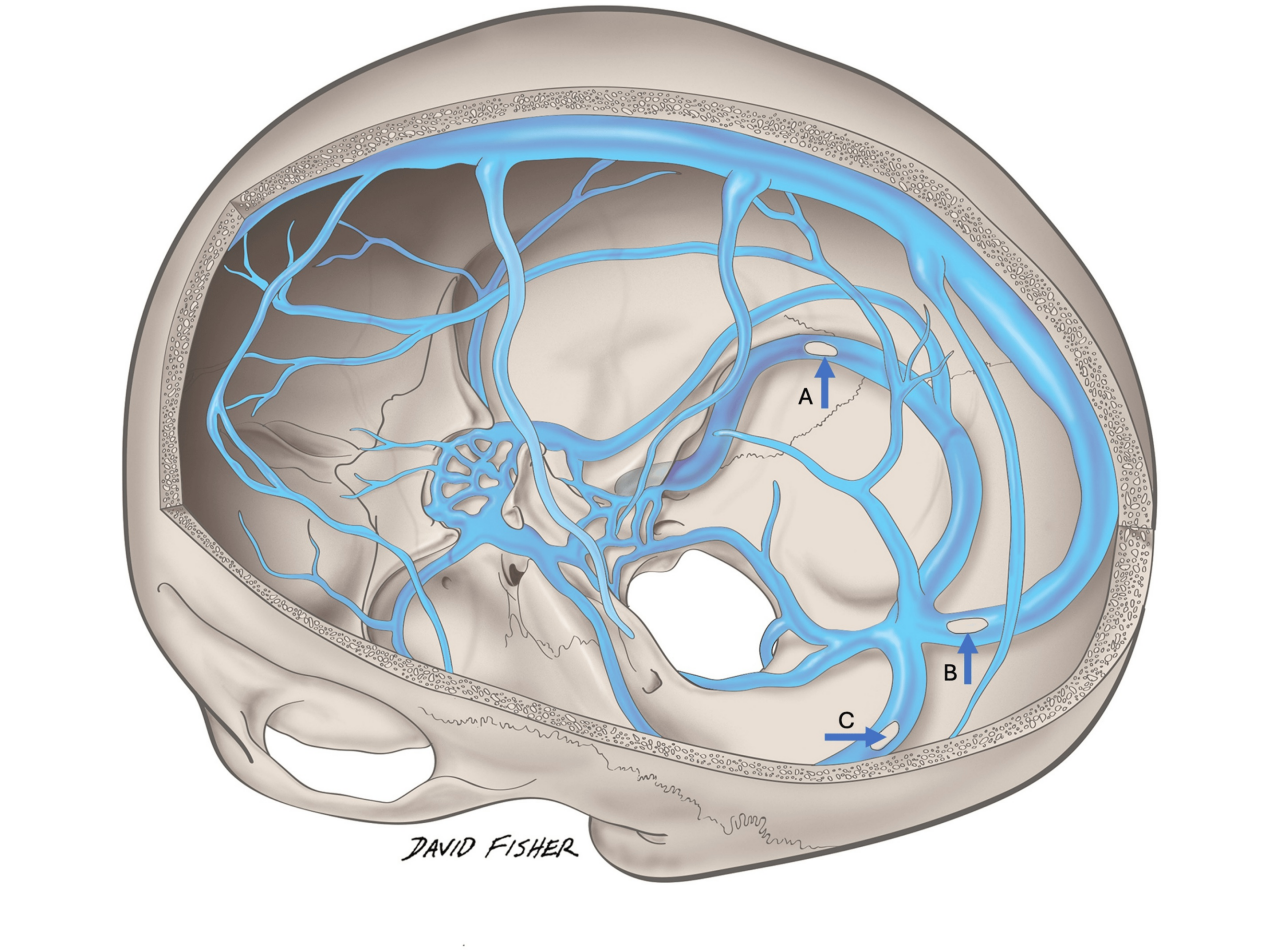
Schematic drawing of the fenestrated sinuses in the present case. Note the fenestrations in the right transverse sinus (A), superior sagittal sinus (B), and left transverse sinus (C) (Courtesy Professor R. Shane Tubbs).

Based on an understanding of the dural venous sinuses' historical embryological development, Figure 1 is illustrated. During the routine dissection of a latex-injected male cadaver, defects in some of the dural venous sinuses were identified. The cadaveric donor was 78 years old at death and died of cardiopulmonary arrest. No intracranial gross pathology was identified, but fenestrations were noted during dissection of the superior sagittal sinuses and both left and right transverse (Figures 2-3). For the superior sagittal sinus, the fenestration was located just proximal to the junction of left and right transverse sinuses and measured 6 cm in length. For the left transverse sinus, the fenestration was located at approximately the midpoint of the sinus and measured 4.5 cm in length. For the right transverse sinus, the fenestration was found just proximal to the junction with the sigmoid sinus and measured 7 cm long. The right transverse sinus was more superiorly located compared to its left counterpart. No occipital sinus was identified, and the straight sinus drained near the proximal left transverse sinus. The right transverse sinus was dominant and slightly larger in diameter throughout its course. The present study was performed in accordance with the requirements of the Declaration of Helsinki (64th WMA General Assembly, Fortaleza, Brazil, October 2013).

## Discussion

To the best of our knowledge, cases of multi-dural venous sinus fenestrations, i.e., fenestrated superior sagittal sinus and bilateral fenestrated transverse sinuses, have yet to be reported in the literature. Cases of a single fenestrated sinus and other anatomical variations of the dural venous sinuses have been well-reported. Massrey et al. [9] and McComiskey and Glikstein [10] each reported a case of an incidental fenestrated left transverse sinus located within 3 cm of the torcular Herophili. Several cases of a fenestrated superior sagittal sinus associated with parietal cephalocele have been reported in infants, often with additional sinus anomalies [11-13]. Kouzmitcheva et al. reported the incidences of hypoplastic sinuses, persistent fetal sinuses, and duplicated/fenestrated/septated sinuses when they investigated the association between these anatomical venous variants and cerebral sinovenous thrombosis in children [14]. In their examination of 131 cadaveric specimens, Browder et al. identified fenestrated and duplicated straight sinuses, variations in the junction of the straight sinus with the torcular Herophili and associated sinuses, and variations in the tributaries coursing through the tentorium cerebelli to join the straight sinus [15]. In a case perhaps most similar to the one discussed here, Rădoi et al. presented a 64-year-old female with an aplastic right transverse sinus, a blind-ending hypoplastic left transverse sinus, and a fenestrated superior sagittal sinus that drained through enlarged occipital and marginal sinuses into the sigmoid sinuses [16]. Rapid increases and decreases in the caliber of the transverse sinuses frequently produce sinuses with irregular diameter, irregular margins, missing segments, septations, and other potential malformations [5]. This ballooning of transverse sinuses can extend into portions of the superior sagittal and superior petrosal sinuses, suggesting that malformations of those sinuses could have a similar origin [5].

## Conclusions

It is rare to find multiple dural venous sinus fenestrations in the same sample. The cause may be the embryological development of the dural venous sinus. The variation in the dural venous system is essentially a rule rather than an exception. Knowledge of the anatomical variation is critical for precluding complications during endovascular or neurosurgical procedures.
